# Obituary

**Published:** 1868-09

**Authors:** 


### Obituary.

DEATH OF DR. GEORGE JAY SWEET.—We are pained to announce the death of Dr. G. J. Sweet, who died at his father's residence in Buffalo, August 12th, 1868, of consumption, in the 36th year of his age.

Dr. Sweet was born in Oppenheim, Montgomery county, N. Y., in 1832, and graduated from the University of New York in the fall of 1854. He removed to Pontiac, Ill., in 1857, where he continued to practice his profession until 1861, when he was employed as assistant surgeon in the U. S. Navy; afterwards acting as fleet surgeon at Key West, Fla. In 1863 he was transferred to the Marine Hospital, Key West, which position he held at the time of his death. Dr. Sweet, though young, gave promise of great usefulness in his profession. His remains were taken to Hume, Allegany county, for interment.

